# Ressourcenbedarf und telemedizinisches Potenzial im Ärztlichen Bereitschaftsdienst: Eine explorative Befragung unter Ärzten

**DOI:** 10.1055/a-2745-8944

**Published:** 2026-01-12

**Authors:** Johannes Hagelskamp, Kalina Witt, Edgar Steiger, Sebastian Carnarius, Dominik Stillfried

**Affiliations:** 1 Zentralinstitut für die kassenärztliche Versorgung in der Bundesrepublik Deutschland, Berlin, Germany

**Keywords:** Ärztlicher Bereitschaftsdienst, Bereitschaftspraxen, Fahr- und Hausbesuchsdienst, Telefonische Beratung, Telemedizin, Out-of-hours Care, After-Hours Care, Home visits, Remote Consultation, Telemedicine

## Abstract

**Hintergrund:**

Der Ärztliche Bereitschaftsdienst (ÄBD) ist Teil des gesetzlichen Sicherstellungsauftrags der Kassenärztlichen Vereinigungen (KVen) und gewährleistet die ambulante Versorgung außerhalb regulärer Sprechstundenzeiten. Trotz koordinierter Erreichbarkeit über die Rufnummer 116117 bestehen strukturelle und qualitative Unterschiede in der Ausgestaltung des ÄBD, insbesondere im Hinblick auf apparative Ausstattung und personelle Ressourcen. Vor dem Hintergrund zunehmender Erwartungen an eine sektorenübergreifende Versorgung und dem bestehenden Fachkräftemangel stellt sich die Frage, welche im ÄBD fehlenden Ressourcen zu vermeidbaren Notaufnahmeeinweisungen führen und inwieweit telemedizinische Optionen aus ärztlicher Sicht zur Entlastung beitragen können.

**Methodik:**

Die Datenerhebung erfolgte zwischen 01.06.2023–31.08.2023 mittels einer anonymisierten online Fragebogenerhebung nach Einladung durch die KVen. Der Fragebogen umfasste Angaben zu demografischen und beruflichen Charakteristika, zum Ressourcenbedarf im ÄBD sowie der Nutzung und dem Potenzial von telemedizinischen Angeboten im ÄBD. Die Auswertung erfolgte deskriptiv und analytisch.

**Ergebnisse:**

Insgesamt nahmen 4052 im ÄBD tätige Ärzte teil. Fehlende Ressourcen, die eine Einweisung zur Folge haben, waren Labordiagnostik, Sonografie und eine andere Fachexpertise. Das höchste Potenzial für telemedizinische Angebote sahen die Ärzte für die fachliche Rücksprache zu Erkrankungsbildern und Therapien sowie der radiologischen Befundung. Knapp 67% der Ärzte gaben an, dass Hausbesuche durch nichtärztliches Personal mit telemedizinischer Unterstützung umsetzbar wären.

**Schlussfolgerung:**

Der gezielte Einsatz telemedizinischer Anwendungen und der Zugang zu diagnostischen Ressourcen können aus Sicht der befragten Ärzte den ÄBD stärken und unnötige Einweisungen vermeiden. Besonders bei fachlichen Konsultationen, Diagnostik und der Unterstützung nichtärztlicher Hausbesuche sehen sie großes Potenzial – auch als Beitrag zur Lösung des Fachkräftemangels. Voraussetzung ist jedoch eine differenzierte Implementierung unter Berücksichtigung rechtlicher, technischer, personeller und finanzieller Rahmenbedingungen.

## Abstract (en)

**Background:**

Our-of-hours care is part of the statutory mandate of the Associations of Statutory Health Insurance Physicians (KVs) and guarantees outpatient care outside of regular consultation hours. Despite coordinated availability via the 116117 telephone number, there are structural and qualitative differences in the implementation of the out-of-hour care, particularly with regard to equipment and human resources. Against the background of increasing expectations of cross-sectoral care and the existing shortage of specialists, the question arises as to which lack of resources in the out-of-hours care leads to avoidable emergency admissions and to what extent telemedical options can help to relieve the burden from a medical perspective.

**Methodology:**

A survey was conducted among physicians on out-of-hours care between June 1st, and August 31st 2023 using an anonymized online questionnaire. Participants were recruited by the Regional Statutory Health Insurance Physician Associations which organize out-of-hours care regionally. The questionnaire included demographic and professional items, questions about resource requirements and potential of telemedical services in the out-of-hours care. Data were subjected to descriptive analysis.

**Results:**

A total of 4,052 physicians participated in the survey. Resource limitations most frequently associated with potentially avoidable hospital admissions were laboratory diagnostics, sonography and specialist expertise. The greatest perceived potential for telemedicine services lay in specialist consultation for diagnostic and therapeutic, as well as for radiological assessment. Approximately 67% of respondents indicated that home visits by healthcare assistants could be supported with telemedical support.

**Conclusion:**

Targeted use of telemedicine and improved access to diagnostic resources can, in the view of the surveyed physicians, strengthen the out-of-hours care and help avoid unnecessary hospital admissions. There is particular potential in specialist consultations, diagnostics, and the support of non-physician home visits—also as a response to the growing shortage of healthcare professionals. However, this requires careful implementation that takes legal, technical, personnel, and financial factors into account.

## Hintergrund

Der gesetzliche Sicherstellungsauftrag der Kassenärztlichen Vereinigungen (KVen) umfasst gemäß § 75 Abs. 1b SGB V die vertragsärztliche Versorgung auch während der sprechstundenfreien Zeiten der Arztpraxen. Die KVen richteten dazu einen Ärztlichen Bereitschaftsdienst (ÄBD) ein, welcher durch Akutleitstellen, unter der Rufnummer 116117 oder online 24/7, koordiniert wird. Während der fahrende Dienst die gesamte sprechstundenfeie Zeit abdeckt, sind die Bereitschaftspraxen, meist nur zu Tagesrandzeiten besetzt. Grundsätzlich sind alle niedergelassenen Ärzte aufgrund ihrer Tätigkeit zur Teilnahme am ÄBD verpflichtet. Jedoch nehmen die KVen diese Verpflichtung unterschiedlich wahr. Darüber hinaus sind die Handlungsmöglichkeiten einen Qualitätsstandard durchzusetzen insofern eingeschränkt, dass Vertragsärzte, aufgrund ihrer Selbständigkeit, nicht weisungsgebunden sind 1 und zugleich, naturgemäß ihrer Spezialisierung, unterschiedliche Kompetenzen aufweisen. Derzeit steigen die Erwartungen des Gesetzgebers an die Leistungsfähigkeit der Patientenservicestellen (116117) und den ÄBD 2 . Aktuell gewährleistet die bisherige apparative und personelle Ausstattung des ÄBDs aus Sicht der Krankenhäuser keine ausreichend verlässliche Arbeitsteilung, um Vorhalteaufwendungen in Notaufnahmen für vermeidbare Inanspruchnahmen der Notfallversorgung reduzieren zu können 3 .

Telemedizinische Angebote der KVen entlasten den ÄBD in den letzten Jahren zunehmend. Daneben haben die Bereitschaftspraxen den Bedarf für Hausbesuche deutlich reduziert 4 . Entwicklungen in der stationären Versorgung zeigen, dass vernetzte Strukturen durch Teleintensivmedizin peripheren Einrichtungen ermöglichen auf zentral vorgehaltene Ressourcen mit spezifischer medizinischer Qualifikation zuzugreifen 5 6 und die Effizienz am Standort steigert 7 .

Da intersektorale Zusammenarbeit unter Bedingungen des Fachkräftemangels im Gesundheitswesen möglichst ressourcenschonend gestaltet werden muss, sollte im Rahmen der vorliegenden Arbeit durch eine Befragung der im ÄBD tätigen Ärzte geprüft werden, welche fehlenden Ressourcen im ÄBD zu potenziell vermeidbaren Einweisungen in Notaufnahmen führen und welches Potenzial für eine stärkere Nutzung telemedizinischer Gestaltungsoptionen besteht, um solche Einweisungen zu vermeiden.

## Methodik

Die vorliegende Studie berichtet nach den „Checklist for Reporting of Survey Studies“ (CROSS) Kriterien 8 .

### Datenerhebung

Vom 01. Juni bis 31. August 2023 wurde eine anonyme online gestützte Fragebogenerhebung auf der Plattform LimeSurvey Version 5 als Querschnittstudie mit Convenience Sampling durchgeführt. Adressaten der Erhebung waren im ÄBD tätige Ärzte in 13 KVen. Die Einladung zur Teilnahme erfolgte per E-Mailverteiler über die beteiligten KVen. Einige KVen führten gezielte Nachfassmaßnahmen durch wiederholtes Versenden von E-Mails mit Aufforderung zur Teilnahme durch. Eine Mehrfachteilnahme wurde durch Cookie Setzung verhindert.

### Studienpopulation

In die Studie eingeschlossen wurden Ärzte aller Geschlechter und Altersgruppen, die in den teilnehmenden KV-Bereichen aktiv im ÄBD tätig sind. Ausgeschlossen wurden Ärzte, die von der Teilnahme am ÄBD befreit sind.

### Fragebogen

In Zusammenarbeit mit den beteiligten KVen wurde ein Fragebogen entwickelt (siehe Online-Supplement). Dieser umfasst 18 Fragen (zzgl. 4 möglicher Unterfragen) gegliedert nach demografischen und beruflichen Charakteristika, Ressourcenbedarf im ÄBD sowie der Nutzung und dem Potenzial von telemedizinischen Angeboten im ÄBD. Es wurde nach Alter und Geschlecht der Fachrichtung und der üblichen Tätigkeit sowie Tätigkeitsdauer, -bereich und Leitungsfunktion im ÄBD gefragt. Darüber hinaus wurde die Einschätzung bezüglich der Ursachen von vermeidbaren Einweisungen in Zusammenhang mit fehlendem Ressourcenzugang zu Labordiagnostik, radiologischer Diagnostik und Konsilen in fachfremden Gebieten eingeholt. Des Weiteren erfolgten Angaben zu unlängst genutzten telemedizinischen Angeboten. Abschließend wurde die Einschätzung zum Potenzial von telemedizinischen Tätigkeitsbereichen, von telemedizinischer Unterstützung bei Hausbesuchen sowie grundsätzlich im ÄBD eingeholt. Erhoben wurden die Antworten anhand von Single-Choice-, Multiple-Choice- und Freitextfeldern. Der Fragebogen wurde in einem Pretest mit vier im ÄBD tätigen Ärzten unterschiedlicher Fachrichtungen und Berufserfahrungen getestet.

### Auswertung

Die Datenauswertung erfolgte mithilfe der Statistik-Software R 9 . Signifikanztests wurden zum Signifikanzniveau von 0,05 durchgeführt. Die Antworten wurden zunächst deskriptiv beschrieben und getrennt nach aggregierten Fachgruppen (Allgemein-/Innere Medizin, Pädiatrie, Sonstige) ausgewertet. Freitextantworten wurden gesichtet. Die vierstufige Likert-Skala („Sehr hoch“/„Eher hoch“/„Eher niedrig“/„Sehr niedrig“) wurde in der Auswertung in zwei Stufen zusammengefasst („Hoch“/„Niedrig“), um die Komplexität für statistische Tests zu reduzieren. Für Vergleiche zwischen verschiedenen Gruppen wurden der Chi-Quadrat-Test mit Bonferroni-Holm-Korrektur und der Kruskal-Wallis-Test angewandt.

### Datenschutz

Die Teilnahme an der Fragebogenerhebung war nach schriftlicher Aufklärung und Einwilligung möglich. Die EU-Datenschutzgrundverordnung, das Berliner Datenschutzgesetz und die Deklaration von Helsinki in ihrer aktuellen Fassung wurden berücksichtigt.

## Ergebnisse

Im Studienzeitraum wurde der Fragebogen 5677 mal aufgerufen. 4052 Ärzte nahmen abschließend an der Befragung teil ( Tab. 1 ).

**Table:** **Tab. 1** Charakteristika der Befragten

Charakteristikum	n	Anteil in %
**Alter (n=4043)**		
Unter 30 Jahre	4	0,1
30 bis 39 Jahre	434	10,7
40 bis 49 Jahre	996	24,6
50 bis 59 Jahre	1359	33,6
60 bis 69 Jahre	1032	25,5
Über 70 Jahre	218	5,4
**Geschlecht (n=4036)**		
Divers	13	0,3
Männlich	2308	57,2
Weiblich	1715	42,5
**Fachrichtung (n=3838)**		
Allgemeinmedizin	1634	40,3
Innere Medizin	648	16,0
Pädiatrie	433	10,7
Gynäkologie	154	3,8
Augenheilkunde	134	3,3
Orthopädie	126	3,1
Chirurgie	117	2,9
Hals-Nasen-Ohren Heilkunde	90	2,2
Neurologie	75	1,9
Urologie	63	1,6
Kardiologie	43	1,1
In Weiterbildung	22	0,5
Sonstiges	299	7,4
**Berufserfahrung (n=4037)**		
Unter 1 Jahr	5	0,1
1 bis 3 Jahre	13	0,3
4 bis 5 Jahre	67	1,7
6 bis 10 Jahre	331	8,2
Über 10 Jahre	3621	89,7
**Haupttätigkeit (n=3876)**		
Niedergelassen	3119	77,2
Angestellt in einer Vertragsarztpraxis	302	7,5
Angestellt in einem Klinikum	190	4,7
Im Ruhestand	155	3,8
Honorararzt	100	2,5
Ohne Tätigkeit	10	0,2
**Bereich im ÄBD (n=3979)**		
Fahr-/Hausbesuchsdienst	1237	31,1
Fahr-/Hausbesuchsdienst mit fachspezifischem Schwerpunkt (HNO/Augenheilkunde etc.)	16	0,4
Telefonischer Beratungsdienst	45	1,1
Bereitschaftspraxen	2053	51,6
Bereitschaftspraxen für Kinder und Jugendliche	448	11,3
Bereitschaftspraxen mit fachspezifischem Schwerpunkt (HNO-/Augenheilkunde etc.)	180	4,5
**Leitungsfunktion (n=4026)**		
Ja	397	9,9
Nein	3629	90,1

ÄBD: Ärztlicher Bereitschaftsdienst, HNO: Hals-Nasen-Ohren

### Fehlende Ressourcen im ÄBD

Auf die Frage nach fehlenden Ressourcen, die potenziell vermeidbare Einweisungen in die Notaufnahme verursachen, gaben 50,5% (n=4052) der Teilnehmer an, dass ihnen Labordiagnostik fehlt. Sonographie (n=4052; 44,6%), fachfremde Beratung (n=4052; 40,6%) und fehlende Röntgendiagnostik (n=4052; 37,2%) sind weitere Restriktionen. Am seltensten wurden CT- (n=4052; 25,1%) und MRT-Untersuchungen (n=4052; 21,5%) als fehlende Ressource erachtet. Die tiefergehende Analyse nach Fachgruppen ergab signifikante Unterschiede in der Einschätzung, welche Ressourcen im ÄBD zur Verhinderung von Einweisungen notwendig wären ( Abb. 1 ). Allgemeinmediziner und Internisten (n=2282) gaben im Vergleich zu anderen Fachgruppen (n=1337), signifikant häufiger einen fehlenden Zugang zu Labor, Sonographie und Röntgen als fehlende Ressource an. Im Vergleich zu Pädiatern (n=433) fehlten Allgemeinmedizinern und Internisten häufiger Bildgebung (CT, Röntgen, Sonographie), Labor und Beratung durch andere Disziplinen. Pädiatern fehlten im Vergleich zu anderen Fachgruppen häufiger die Befundung von Röntgen und CT-Untersuchungen sowie fachfremde Beratung anderer Fachgruppen.

**Abb. 1:**
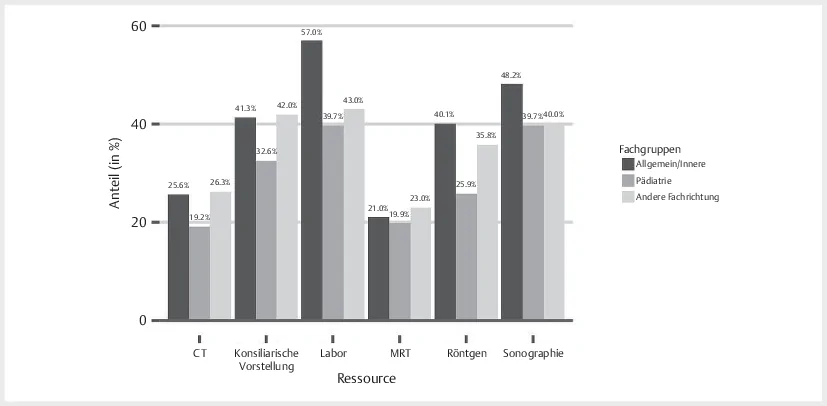
Fehlende diagnostische Ressourcen je Fachgruppe, die eine Einweisung in die Notaufnahme erforderlich machen. n=4052, Mehrfachauswahl. CT-Computertomographie; MRT-Magnetresonanztomographie

Befragte, die einen fehlenden Zugang zu Laborressourcen dokumentierten (n=2048), gaben am häufigsten an, dass ein kleines Blutbild (76,8%), Troponin-T-Nachweis (67,9%), D-Dimere (66,6%) oder eine Blutchemieuntersuchung (61,0%) für ihre Tätigkeit im ÄBD fehlten.

Die Ärzte konnten angeben, welche Fachrichtungen bei telemedizinischer Rücksprache erforderlich wären. Am häufigsten wurde über alle Fachgruppen hinweg (n=4.052) die Vorhaltung von neurologischen (41,4%) und pädiatrischen (37,1%) Konsilen angegeben. Am seltensten wurden allgemeinmedizinische (15,2%), urologische (22,4%) und internistische (23,4%) Konsile als notwendig erachtet. Unter den Allgemeinmedizinern/Internisten (n=2282) wurde der Bedarf an Rücksprache für Neurologie (47,6%), Pädiatrie (41,0%) und Augenheilkunde (37,9%) am häufigsten gewählt. Unter den weiteren Fachrichtungen (n=1337) wurde am häufigsten ein Bedarf an telemedizinischer Rücksprache mit Neurologen (38,8%), Pädiatern (38,1%), Internisten (36,0%) und Kardiologen (35,9%) gesehen.

### Telemedizinisches Potenzial im ÄBD

Im Kontext des telemedizinischen Potenzials im ÄBD wurde zunächst der aktuelle Nutzungsgrad (n=4052) telemedizinischer Angebote erfragt. Am häufigsten wurde die Labordatenübermittlung angegeben, die von 10,1% der Teilnehmenden genutzt wird. Diagnostische Geräte mit Möglichkeit zur Fremdbefundung nutzen bereits 5,2%, die Unterstützung zur fachfremden Befundung 4,6% und Videokonsultation 4,1%. Eine telemedizinische Befundung von Röntgendiagnostik (1,8%) und Sonografie (0,7%) kamen am seltensten zum Einsatz.

Das höchste Potenzial für telemedizinische Anwendungen sahen die Befragten (n=4052) bei der fachlichen Rücksprache für fachfremde Erkrankungsbilder (71,6%) oder fachfremde Therapien (70,6%) sowie die Befundung von radiologischer Diagnostik (71,3%). Am niedrigsten wurde das Potenzial für Auskultation (80,0%), urologische/gynäkologische Untersuchungen (73,6%) und Untersuchungen am Bewegungsapparat (72,6%) eingeschätzt.

Die Ärzte gaben zu 66,9% (n=3718) an, dass Hausbesuche durch nicht ärztliches Personal telemedizinisch unterstützt werden könnten. Signifikante Unterschiede zeigten sich zwischen den Einsatzbereichen im ÄBD: Ärzte in einer Bereitschaftspraxis waren zu 68,7% (n=1117) der Meinung, dass die telemedizinische Unterstützung von Hausbesuchen sinnvoll ist. Ärzte im Fahr- und Hausbesuchsdienst teilten diese Auffassung zu 62,6% (n=1117).

Insgesamt 64,9% (n=3624) aller Befragten schätzen den Anteil der potenziell telemedizinischen Behandlungsfälle im ÄBD auf unter 20%. Im Median lag der Anteil bei 15% und im arithmetischen Mittel bei 22,3%. Ärztinnen gaben mit durchschnittlich 24,5% ein signifikant (n=3613) höheres Potenzial für an als Ärzte (20,8%). Beim Alter (n=3620), den Fachgruppen (n=3624) und den Haupttätigkeiten (n=3616) zeigten sich ebenfalls signifikante Unterschiede. Ärzte der Altersgruppe 30–39 Jahre sahen mit 28,9% das höchste Potenzial, während die 60–69-Jährigen es mit 18,8% am niedrigsten einschätzten. Orthopäden (31,3%), Neurologen (29,1%) und Gynäkologen (28,0%) schätzten das Potenzial am höchsten ein, wohingegen die Pädiater (17,3%), Augenärzte (20,0%) und Urologen (20,5%) die niedrigsten durchschnittlichen Bewertungen angaben. Angestellte Ärzte im Klinikum (29,2%) oder der Vertragsarztpraxis (24,2%) schätzen den Bedarf am höchsten ein. Das niedrigste Potenzial sahen Ärzte im Ruhestand (16,3%), Honorarärzte (21,5%) und Niedergelassene (22,1%).

## Diskussion

### Digitalisierung im Gesundheitswesen

Die Integration telemedizinischer Anwendungen würde nicht nur die Effizienz steigern, sondern auch Versorgungsengpässe in peripheren Regionen entschärfen können 10 . Ein zentrales Ergebnis der vorliegenden Befragung ist der hohe Bedarf an telemedizinischen Konsilien, insbesondere in den Bereichen Neurologie (41,4%) und Pädiatrie (37,1%). Diese Ergebnisse stehen im Einklang mit Studien, die zeigen, dass Pädiatrie und Neurologie häufig komplexe und fachspezifische Beratungen erfordern, die durch telemedizinische Lösungen unterstützt werden können 11 12 . Gleichzeitig zeigt der geringe Nutzungsgrad von telemedizinischer Rücksprache für Allgemeinmedizin und Urologie (15,2% bzw. 22,4%), dass der Bedarf an telemedizinischer Unterstützung stark von der Fachrichtung abhängt. Erste wissenschaftliche Erkenntnisse aus Deutschland belegen, dass bei telemedizinischer Unterstützung keine signifikanten Unterschiede in der Diagnosegenauigkeit zwischen vor Ort tätigen Notärzten und Telenotärzten bestehen, insbesondere bei nicht akut lebensbedrohlichen Fällen 13 14 . Es zeigt sich zudem, dass die Notarztquote bei Einsätzen von Rettungswägen mit telemedizinischer Konsultationsoption um mehr als 20% gesenkt werden kann 15 . Im ländlichen Raum ergeben sich zusätzliche Effizienzgewinne durch die Einsparung von Fahrtzeiten, was die Verfügbarkeit von ärztlichem Personal erhöht, und die Ressourcenverteilung verbessert 16 .

### Fehlende Ressourcen, diagnostische Einschränkungen im ÄBD und telemedizinisches Potenzial

Fehlende Labordiagnostik (50,5%), Sonographie (44,6%) und Röntgendiagnostik (37,2%) werden von den Ärzten als Gründe für vermeidbare Einweisungen in die Notaufnahmen angegeben. Diese Ergebnisse spiegeln Erkenntnisse aus anderen Studien wider, die zeigen, dass der Zugang zu diagnostischen Verfahren essenziell ist, um unnötige Krankenhausaufenthalte zu vermeiden 17 . Besonders hervorzuheben ist der Bedarf an spezifischen Laborparametern wie D-Dimeren (66,6%) und Troponin-T (67,9%), die entscheidend für die Differenzialdiagnose akuter Erkrankungen sind. Mobile Diagnostikeinheiten (insbesondere Sonographie), point-of-care-Labore oder die Nutzung vorhandener Infrastruktur in nahegelegenen Kliniken oder ÄBD-Praxen könnten Lösungsansätze bieten, um telemedizinische Konsile sinnvoll zu ergänzen.

Diese Ergebnisse unterstreichen die Notwendigkeit, telemedizinische Diagnostik in den ÄBD zu integrieren, um zeitkritische Entscheidungen zu erleichtern. Die Befragung zeigt, dass telemedizinische Anwendungen wie fachliche Rücksprachen (71,6%) und radiologische Befundungen (71,3%) von den Ärzten als besonders vielversprechend eingeschätzt werden. Dies stimmt mit der Literatur überein, die aufzeigt, dass Telekonsile und radiologische Ferndiagnostik bereits erfolgreich implementiert wurden und die Versorgungsqualität signifikant verbessern können 18 . Der geringe Einsatz telemedizinischer Technologien wie Videokonsultationen (4,1%) und telemedizinischer Sonographien (0,7%) deutet jedoch darauf hin, dass technische und organisatorische Barrieren bestehen. Diese Diskrepanz zwischen Potenzial und aktueller Nutzung erfordert weitere Forschung und Investitionen, um die Akzeptanz und Implementierung zu verbessern. Eine erste Studie für ein sektorenübergreifendes Versorgungskonzept zeigt ein hohes Potenzial 19 . Die telemedizinische Diagnostik hat sich in einigen Anwendungsfeldern bereits bewährt, etwa bei der Befundung von EKGs 13 und dermatologischen Untersuchungen 20 .

### Bisherige Erfahrungen mit dem Einsatz telemedizinischer Komponenten in der Akut- und Notfallversorgung

Die Inanspruchnahme der Notfallstrukturen geht zu erheblichen Teilen auf Personen ohne dringlichen Versorgungsbedarf zurück 21 22 23 . Die Entlastung der Notaufnahmen um ambulant behandelbare Fälle zu den Zeiten des ÄBD könnte durch eine gesteigerte Nutzung telemedizinischer Möglichkeiten verbessert werden. Darüber hinaus könnte ein verbesserter Zugang zu Labordiagnostik, Sonographie und fachfremder Beratung, die am häufigsten genannten Restriktionen mit Einweisungsfolge reduziert werden. So könnten telemedizinische Konsile in den Bereichen Neurologie, Pädiatrie und Radiologie die Diagnostik und Behandlung im ÄBD unterstützen. Zudem könnten Hausbesuche durch nicht ärztliches Personal mithilfe telemedizinischer Hilfsmittel effizient und qualitativ hochwertig durchgeführt werden 15 . In einem Modellprojekt, bei dem examinierte Gesundheitsfachkräfte mithilfe telemedizinischer Beratung Hausbesuche bei Akutfällen im ÄBD durchführten, konnte in 63,5% der Fälle eine abschließende Versorgung sichergestellt werden. Die Patienten zeigten eine sehr hohe Akzeptanz dieser Versorgungsform (94,5%) 24 . 66,9% der Befragten erachten die telemedizinische Unterstützung nicht ärztlicher Fachkräfte bei Hausbesuchen als sinnvoll. Allerdings wäre dies mit einem grundlegenden Umbau der medizinischen Versorgung zu Bereitschaftsdienstzeiten verbunden. Möglichkeiten der Delegation an nicht ärztliche Fachberufe in der Peripherie könnten durch ein breiteres Spektrum fachärztlicher Kompetenz im Wege der Telemedizin erweitert und unterstützt werden. Projekte, wie das Telenotarztsystem und die telemedizinisch unterstützten Gesundheitsfachkräfte bei Hausbesuchen in Niedersachsen zeigen, dass die Kombination aus technischer Unterstützung und menschlicher Expertise ein zukunftsfähiges Modell für die Gesundheitsversorgung darstellt 24 25 . Darüber hinaus erscheint es sinnvoll, auch organisatorische Maßnahmen wie eine verbesserte Vernetzung zwischen ÄBD, Rettungsdienst und Notaufnahmen sowie den Einsatz strukturierter Ersteinschätzungsverfahren (z. B. Strukturierte medizinische Ersteinschätzung in Deutschland (SmED)) zu stärken, um Patientenströme gezielter zu steuern 26 . Langfristig könnten Notdienstpraxen oder integrierte Notfallzentren (INZ) an Krankenhausstandorten eine zentrale Rolle spielen, indem sie Triagefunktion, ambulant nutzbare diagnostische Infrastruktur und telemedizinische Expertise bündeln. Dies könnte die Versorgungseffizienz erhöhen, sofern eine Fokussierung der Inanspruchnahme auf diese Standorte erfolgt. Zudem erfordert die Umsetzung telemedizinischer Konsile eine koordinierte Struktur, die digitale Konsile, dezentrale Bereitschaftspraxen, mobile Einsatzteams und telemedizinische Unterstützung integriert. Modellprojekte wie in Niedersachsen 24 25 verdeutlichen, dass ein solches mehrstufiges, vernetztes System sowohl die Versorgungsqualität als auch die Ressourcennutzung verbessern kann.

Gleichzeitig zeigt sich, dass fehlende Labor- und Bildgebungskapazitäten sowie organisatorische Barrieren nach wie vor zentrale Limitierungen darstellen. Eine nachhaltige Verbesserung der Versorgung erfordert daher nicht nur die Implementierung digitaler Konsile und telemedizinischer Hausbesuche, sondern auch die gezielte Erweiterung diagnostischer Ressourcen sowie die Integration der Telekonsile in organisatorische Strukturen der Akutversorgung, die räumlich begrenzte Ressourcen wie Labor und radiologische Bildgebung vorhalten. Zukünftige Studien sollten untersuchen, wie eine integrierte Versorgungsstruktur – bestehend aus telemedizinischer Unterstützung, dezentralen ÄBD-Praxen, mobilen Einsatzteams und strukturierten Ersteinschätzungsverfahren – die Einweisungszahlen und die Versorgungsqualität im ÄBD beeinflussen kann. Ein solcher Forschungsfokus könnte dazu beitragen, evidenzbasierte Empfehlungen für die Weiterentwicklung des ÄBD zu formulieren und die Versorgungssituation für Patientinnen und Patienten langfristig zu verbessern.

### Stärken und Limitationen

Die vorliegende Studie stellt die erste Untersuchung ihrer Art dar, die sich speziell mit den im ÄBD tätigen Ärzten befasst und auf fehlende Ressourcen und ergänzende telemedizinischen Potenziale hinweist. Bislang existieren in der Literatur keine vergleichbaren Befragungen, wodurch ein direkter Vergleich oder eine Validierung der Ergebnisse erschwert wird.

Aufgrund von Limitationen, die im Untersuchungsgegenstand selbst und im Zugang zu den im ÄBD tätigen Ärzten liegen, kann dieser Untersuchung nur ein explorativer Charakter zukommen. Ein Grund liegt in der Heterogenität der Regelungen zur Dienstverpflichtung im ÄBD. Hier variieren die Anforderungen stark zwischen den Bundesländern 27 . Häufig wird der ÄBD durch sogenannte Pool- oder Honorarärzte ergänzt. Diese regionalen Unterschiede sowie der hausärztlich/internistische Schwerpunkt in den Bereitschaftspraxen zur Erwachsenenversorgung erschweren eine präzise Erfassung und spiegeln sich auch in der Befragung wider. Allgemeinmediziner und Internisten sind in den Ergebnissen überrepräsentiert, während andere Fachgruppen möglicherweise unterrepräsentiert sind. Dies könnte zu einer Verzerrung der Wahrnehmung bezüglich fehlender Ressourcen führen. Auch aufgrund des indirekten Zugangs zu den Teilnehmenden kann keine Repräsentativität im statistischen Sinne hergestellt oder angenommen werden. Zwar richtete sich die Befragung grundsätzlich an alle in der vertragsärztlichen Versorgung tätigen Ärzte (n=154630), jedoch ist unklar, welcher Anteil der potenziell erreichbaren Personen durch die jeweiligen KVen erreicht wurde. Hinzu kommt, dass für den Befragungszeitraum keine Gesamtanzahl, der im ÄBD tätigen Poolärzte in den KVen vorliegt. Dennoch ermöglicht die hohe Fallzahl und die überregionale Beteiligung aus 13 KVen eine Betrachtung relevanter Subgruppen.

## Zusätzliches Material

Zusätzliches Material Zusätzliches Material
