# Supplementary material for: Medical resource needs and telemedical potential in after-hours care: An exploratory survey among physicians
Source: Gesundheitswesen. 2026 Jan 12;88(10):585–91. [Article in German] doi: 10.1055/a-2745-8944 (PMC13645418; doi:10.1055/a-2745-8944)
Supplement: Supplementary file 1 [file 10-1055-a-2745-8944-2025-06-2294-oa.pdf]

Befragung zur Anwendung von Telemedizin im Ärztlichen Bereitschaftsdienst

Herzlich Willkommen,

wir danken Ihnen für Ihre Bereitschaft zur Teilnahme an dieser Befragung!

In der folgenden Umfrage möchten wir erfassen, an **welcher Stelle** und in **welchem Umfang** der Bereitschaftsdienst mit **telemedizinischen Anwendungen unterstützt** werden kann.

Die Teilnahme an der Befragung ist freiwillig. Die Bearbeitungsdauer beträgt ca. 10 Minuten. Die Datenerhebung erfolgt anonym und Ihre Antworten können nicht auf Sie zurückgeführt werden. Das bedeutet ebenfalls, dass Ihr persönlicher Datensatz nach Abschluss der Befragung für uns nicht identifizierbar ist. Alle erhobenen Daten sind **anonym** und werden streng vertraulich behandelt.

Die Teilnahme ist bis zum 31.07.2023 möglich.

Bei Fragen oder Anmerkungen kontaktieren Sie gern unser Projekt-Team: medizin@zi.de

In dieser Umfrage sind 22 Fragen enthalten.

Allgemeine Angaben

Das Gesundheitswesen | DOI 10.1055/a-2745-8944 | © The Author(s).

Wie alt sind Sie? *

Bitte wählen Sie nur eine der folgenden Antworten aus:

- ◯ Unter 30 Jahre
- ◯ 30 bis 39 Jahre
- ◯ 40 bis 49 Jahre
- ◯ 50 bis 59 Jahre
- ◯ 60 bis 69 Jahre
- ◯ Über 70 Jahre

Welches Geschlecht haben Sie? *

Bitte wählen Sie nur eine der folgenden Antworten aus:

- ◯ Weiblich
- ◯ Männlich
- ◯ Divers

Angaben zum beruflichen Hintergrund

Das Gesundheitswesen | DOI 10.1055/a-2745-8944 | © The Author(s).

Welche Fachrichtung haben Sie?

Hinweis: Bei mehr als einer Fachrichtung oder einer Zusatzbezeichnung, wählen Sie diejenige, die zur Niederlassung zählt.

Bitte wählen Sie nur eine der folgenden Antworten aus:

- ◯ Allgemeinmedizin
- ◯ Augenheilkunde
- ◯ Chirurgie
- ◯ Gynäkologie
- ◯ Hals-Nasen-Ohren Heilkunde
- ◯ Innere Medizin
- ◯ Kardiologie
- ◯ Neurologie
- ◯ Orthopädie
- ◯ Pädiatrie
- ◯ Urologie
- ◯ In Weiterbildung
- ◯ Sonstiges

Seit wie vielen Jahren sind Sie ärztlich tätig?

*

Bitte wählen Sie nur eine der folgenden Antworten aus:

- ◯ Unter 1 Jahr
- ◯ 1 bis 3 Jahre
- ◯ 4 bis 5 Jahre
- ◯ 6 bis 10 Jahre
- ◯ Über 10 Jahre

Das Gesundheitswesen | DOI 10.1055/a-2745-8944 | © The Author(s).

Sind Sie niedergelassen? *

Bitte wählen Sie nur eine der folgenden Antworten aus:

- ◯ Ja
- ◯ Nein

Wo sind Sie hauptberuflich tätig? *

Beantworten Sie diese Frage nur, wenn folgende Bedingungen erfüllt sind:
G05 == "02"

Bitte wählen Sie nur eine der folgenden Antworten aus:

- ◯ Angestellt in einem Klinikum
- ◯ Angestellt in einer Vertragsarztpraxis
- ◯ Honorarärztin / Honorararzt
- ◯ Im Ruhestand
- ◯ Ohne Tätigkeit
- ◯ Sonstiges

Angaben zum Tätigkeitsbereich im Bereitschaftsdienst

Das Gesundheitswesen | DOI 10.1055/a-2745-8944 | © The Author(s).

In welcher Kassenärztlichen Vereinigung sind Sie tätig? *

Bitte wählen Sie nur eine der folgenden Antworten aus:

- ◯ KV Baden-Württemberg
- ◯ KV Bayerns
- ◯ KV Berlin
- ◯ KV Brandenburg
- ◯ KV Bremen
- ◯ KV Hamburg
- ◯ KV Hessen
- ◯ KV Mecklenburg-Vorpommern
- ◯ KV Niedersachsen
- ◯ KV Nordrhein
- ◯ KV Rheinland-Pfalz
- ◯ KV Saarland
- ◯ KV Sachsen
- ◯ KV Sachsen-Anhalt
- ◯ KV Schleswig-Holstein
- ◯ KV Thüringen
- ◯ KV Westfalen-Lippe
- ◯ Keine Angabe

Das Gesundheitswesen | DOI 10.1055/a-2745-8944 | © The Author(s).

In welchem Bereich des Bereitschaftsdienstes sind Sie tätig? *

Bitte wählen Sie nur eine der folgenden Antworten aus:

- ◯ In Notdienstpraxen / Bereitschaftspraxen / Portalpraxen / Anlaufpraxen
- ◯ In Notdienstpraxen / Bereitschaftspraxen / Portalpraxen / Anlaufpraxen für Kinder und Jugendliche
- ◯ In Notdienstpraxen / Bereitschaftspraxen / Portalpraxen / Anlaufpraxen mit fachspezifischem Schwerpunkt (HNO/Augenheilkunde etc.)
- ◯ Im telefonischen Beratungsdienst / in der Video- und Telekonsultation im Bereitschaftsdienst
- ◯ Im Fahr-/Hausbesuchsdienst
- ◯ Im Fahr-/Hausbesuchsdienst mit fachspezifischem Schwerpunkt (HNO/Augenheilkunde etc.)

Haben Sie eine leitende ärztliche Funktion im Bereitschaftsdienst? *

Bitte wählen Sie nur eine der folgenden Antworten aus:

- ◯ Ja
- ◯ Nein

Angaben zur Telemedizin im Bereitschaftsdienst

Das Gesundheitswesen | DOI 10.1055/a-2745-8944 | © The Author(s).

Welche telemedizinischen Angebote nutzen Sie bereits im Bereitschaftsdienst?

Mehrfachantworten möglich.

Bitte wählen Sie alle zutreffenden Antworten aus:

- ☐ Videokonsultation
- ☐ Diagnostische Geräte mit Möglichkeit zur Fremdbefundung (Digitale RR-Manschette/EKG-Gerät etc.)
- ☐ Telemedizinische Befundung von Röntgenbildern
- ☐ Telemedizinische Befundung von Sonographien
- ☐ Übermittlung von Befunden bei fachfremden Erkrankungen zur konsiliarischen Beurteilung
- ☐ Labordatenübermittlung
- ☐ Sonstiges: ________

Fehlende Ressourcen im Bereitschaftsdienst

Das Gesundheitswesen | DOI 10.1055/a-2745-8944 | © The Author(s).

Welche diagnostische Ressource fehlt Ihnen, die eine Einweisung in die Notaufnahme notwendig macht?

Mehrfachantworten möglich.

*

Bitte wählen Sie alle zutreffenden Antworten aus:

- ☐ Labordiagnostik
- ☐ Röntgendiagnostik
- ☐ CT-Diagnostik
- ☐ MRT-Diagnostik
- ☐ Sonographie
- ☐ Konsiliarische Vorstellung aufgrund einer fachfremden Erkrankung
- ☐ Sonstiges: ______

Das Gesundheitswesen | DOI 10.1055/a-2745-8944 | © The Author(s).

Welche Labordiagnostik fehlt Ihnen?

Mehrfachantworten möglich.

*

Beantworten Sie diese Frage nur, wenn folgende Bedingungen erfüllt sind:
((G13_01.NAOK == "Y"))

Bitte wählen Sie alle zutreffenden Antworten aus:

- ☐ Kleines Blutbild
- ☐ Großes Blutbild
- ☐ Blutchemie
- ☐ Strep-A
- ☐ RSV-Schnelltest
- ☐ D-Dimere
- ☐ Troponin
- ☐ Urindiagnostik
- ☐ Sonstiges:

Wie häufig halten Sie Rücksprache mit anderen Fachrichtungen, um Patientinnen und Patienten vollumfänglich versorgen zu können? *

Bitte wählen Sie nur eine der folgenden Antworten aus:

- ○ Sehr häufig
- ○ Häufig
- ○ Weniger häufig
- ○ Nie

Das Gesundheitswesen | DOI 10.1055/a-2745-8944 | © The Author(s).

Welche Fachrichtungen sollten für telemedizinische Rücksprachen bei fachfremden Erkrankungsbildern vorgehalten werden?

Mehrfachantworten möglich.

*

Bitte wählen Sie alle zutreffenden Antworten aus:

- ☐ Allgemeinmedizin
- ☐ Augenheilkunde
- ☐ Gynäkologie
- ☐ Innere Medizin
- ☐ Kardiologie
- ☐ Neurologie
- ☐ Urologie
- ☐ Pädiatrie
- ☐ Sonstiges: ____________

Das Gesundheitswesen | DOI 10.1055/a-2745-8944 | © The Author(s).

Wie findet bislang die Rücksprache mit anderen Fachrichtungen statt? *

Beantworten Sie diese Frage nur, wenn folgende Bedingungen erfüllt sind:
G15 == "01" or G15 == "02" or G15 == "03"

Bitte wählen Sie eine der folgenden Antworten:
Bitte wählen Sie nur eine der folgenden Antworten aus:

- ◯ Im direkten Gespräch mit den Ärzten und Ärztinnen in der angeschlossenen Notaufnahme
- ◯ In einem Telefongespräch mit den Ärzten und Ärztinnen von einer Station des Krankenhauses
- ◯ In einem kollegialen Telefonat mit mir bekannten Ärzten und Ärztinnen
- ◯ Sonstiges

Telemedizin im Bereitschaftsdienst

Das Gesundheitswesen | DOI 10.1055/a-2745-8944 | © The Author(s).

Wie bewerten Sie das Potenzial der Telemedizin in den nachfolgenden Tätigkeitsbereichen?

*

Bitte wählen Sie die zutreffende Antwort für jeden Punkt aus:

	Sehr hoch	Eher hoch	Eher niedrig	Sehr niedrig	Keine Angabe
**Anamneseerhebung**	○	○	○	○	○
**Auskultation**	○	○	○	○	○
**Ophthalmologische Untersuchung**	○	○	○	○	○
**HNO-Untersuchung**	○	○	○	○	○
**Untersuchung Bewegungsapparat**	○	○	○	○	○
**Neurologische Untersuchung**	○	○	○	○	○
**Dermatologische Untersuchung**	○	○	○	○	○
**Uro-/Gynäkologische Untersuchung**	○	○	○	○	○
**EKG-Befundung**	○	○	○	○	○
**Interpretation von Laborbefunden**	○	○	○	○	○
**Befundung radiologischer Diagnostik**	○	○	○	○	○

Das Gesundheitswesen | DOI 10.1055/a-2745-8944 | © The Author(s).

	Sehr hoch	Eher hoch	Eher niedrig	Sehr niedrig	Keine Angabe
**Fachliche Rücksprache bei unklaren Erkrankungsbildern**	○	○	○	○	○
**Fachliche Rücksprache zur Therapie und Procedere**	○	○	○	○	○

Wo sehen Sie weitere Möglichkeiten für eine telemedizinische Unterstützung? *

Bitte geben Sie Ihre Antwort hier ein:

Sind Sie der Meinung, dass Hausbesuche durch nicht-ärztliches Personal telemedizinisch unterstützt werden könnten? *

Beantworten Sie diese Frage nur, wenn folgende Bedingungen erfüllt sind:
G07 != "02"

Bitte wählen Sie nur eine der folgenden Antworten aus:

- ○ Ja
- ○ Nein

Das Gesundheitswesen | DOI 10.1055/a-2745-8944 | © The Author(s).

Wie hoch schätzen Sie den Prozentsatz an Patientinnen und Patienten, bei denen der Bedarf für eine telemedizinische Unterstützung besteht?

Angabe in Prozent.

*

Ihre Antwort muss zwischen 0 und 100 liegen.
In diesem Feld darf nur ein ganzzahliger Wert eingetragen werden.
Bitte geben Sie Ihre Antwort hier ein:

-

Videokonsultation im Bereitschaftsdienst

Bei welchen Beschwerden stellt der Einsatz von Videokonsultationen insbesondere einen Nutzen dar?

Bitte geben Sie Ihre Antwort hier ein:

Das Gesundheitswesen | DOI 10.1055/a-2745-8944 | © The Author(s).

Welche Vorteile sehen Sie beim Einsatz von Videokonsultationen in der Versorgung von Patientinnen und Patienten gegenüber einer telefonischen Beratung?

Bitte geben Sie Ihre Antwort hier ein:

Was möchten Sie uns noch mitteilen? Hier haben Sie Platz für Fragen und Anmerkungen.

Bitte geben Sie Ihre Antwort hier ein:

Vielen Dank für Ihre Unterstützung!
31.07.2023 – 23:59
Übermittlung Ihres ausgefüllten Fragebogens:
Vielen Dank für die Beantwortung des Fragebogens.

Das Gesundheitswesen | DOI 10.1055/a-2745-8944 | © The Author(s).
